# Direct detection and identification of viruses in saliva using a SpecID ionization modified mass spectrometer

**DOI:** 10.1371/journal.pone.0316368

**Published:** 2025-02-07

**Authors:** Pierre Alusta, Angel Paredes, Marli Azevedo, Lisa Mullis, Dan Buzatu

**Affiliations:** 1 Systems Biology Division, National Center for Toxicological Research (NCTR), U.S. Food and Drug Administration, Jefferson, AR, United States of America; 2 NCTR / ORA Nanotechnology Facility, Office of Scientific Coordination, National Center for Toxicological Research (NCTR), U.S. Food and Drug Administration, Jefferson, AR, United States of America; 3 Division of Microbiology, National Center for Toxicological Research (NCTR), U.S. Food and Drug Administration, Jefferson, AR, United States of America; Cairo University Faculty of Veterinary Medicine, EGYPT

## Abstract

The COVID-19 (SARS-CoV-2) pandemic has led to a significant mortality globally and persistent health challenges in many survivors. Early accurate diagnosis, surveillance, identification of cohorts, and prophylaxis are considered essential measures to reduce the spread of infectious viral pathogens such as SARS-CoV-2. A reliable, fast, high-throughput screening method that can detect viral particles and identify the pathogenic virus in infected individuals could help to reduce the spread of the next viral threat through quick knowledge and implementation of appropriate prevention strategies. Since respiratory viruses are typically present in nasal and oral secretions, saliva is a good target for testing for viral infections. Saliva testing has slowly gained popularity in the diagnostics based on biomarkers and other constituents ranging from organic compounds (*e*.*g*., food additives), peptides, and even microorganisms. Polymerase chain reaction (PCR) remains the gold standard for sensitive detection of SARS-CoV-2 infection in biological samples. However, while PCR testing for COVID is sensitive and widely used by hospitals, the method has a false-negative rate of 15–20% and is kit-based necessitating the development of alternative methods of detection that provide higher accuracy. This paper describes the use of a SpecID Mass Spectrometer that can detect the presence of viral particles in saliva at very low levels (<500 virions/0.5 ml). The main goal of this study was to demonstrate that our previously developed, portable, mass spectrometry based method, SpecID, could also be sued for detecting viruses in saliva, including but not limited to SARS-CoV-2; the SpecID method has the potential to provide a reliable solution that overcomes some of the challenges with molecular testing like PCR.

## 1. Introduction

The COVID-19 (*i*.*e*., SARS-CoV-2) pandemic has been the biggest global health challenge since the Second World War [1], with 679,767,596 confirmed cases and over 6,798,868 mortalities worldwide (WHO report February 2023) [2]. Accurate early diagnosis, monitoring, isolation, and prophylaxis are essential to slowing down the spread of viral threats like SARS-CoV-2 [2, 3]. In order to achieve this, there is a need for a rapid, reliable, high-throughput screening method [4]. A number of analytical laboratory-based methods have been investigated for swift, accurate detection of SARS-CoV-2 [2]. With detection and monitoring of SARS-CoV-2 in mind, Rehman *et al*. explored spectroscopy [5], Connell *et al*. developed a rapid and sturdy IgG-capture enzyme-linked immune-absorbent assay [6], Cabitza *et al*. used blood test analyses and machine learning as an alternative to RT-PCR [7], and Chan *et al*. used TEM (transmission electron microscopy) to identify virus morphology [8]. Genome sequencing is also used to identify viral strains, and the sequence data is necessary for designing PCR primers [9]. Data obtained from smartwatches (*i*.*e*., consumer wearable devices) such as a) time spent resting, b) number of daily steps walked and c) heart rate variations have been evaluated for the detection of COVID-19 at the pre-symptomatic stage [10]. Even PCR, the gold standard technique for detecting SARS-CoV-2 infection in biological samples, yields false-negative rates ranging between 15 and 20% [11–19]. Unfortunately, most of these novel methods have significant limitations [20] and are consequently restricted in their field of applications. Thus, there remains a considerable need for an alternative, accurate, quick, high-throughput screen for viral pathogens, including SARS-Cov-2 variants, that can directly detect viral particles in body fluids (*i*.*e*., saliva, blood, urine) [18, 20–23] readily collected from test subjects in different community settings [5, 24, 25]. Saliva is an easily accessible and reliable biofluid for detecting viral loads, particularly for respiratory viruses like SARS-CoV-2 [16].

### 1.1. Benefits of using saliva

Saliva is a biofluid that is regularly secreted by the salivary glands [25]. It fulfills several functions, including providing a protective coating for the buccal cavity with antimicrobial properties and assisting digestion [1]. Respiratory viruses can easily spread through nasal and oral secretions [26] from infections of the buccal cavity and salivary glands, which leads to subsequent release of particles in saliva via the salivary ducts [26, 27]. Saliva is a readily accessible, easily sampled [1, 13, 27–29] diagnostic bodily fluid [29–31] that can contain infectious agents [32], while preserving high-quality DNA and viral material at room temperature for transport and analysis [18]. In contrast, nasal swabs used in nasopharyngeal sampling are invasive and can be distressing to some patients [1], as it can cause sneezing [33] and bleeding in some cases [1, 33–35]. As a diagnostic biofluid, saliva also has a high consistency rate of > 90% [1]. The practicality and applicability of using nasopharyngeal swabs and saliva to detect viruses has been studied and both are well-accepted for diagnostic testing [17, 33, 34, 36]. An advantage of using a less invasive [15], quick [37] and safe collection [24] of salivary secretion as a substrate for molecular diagnosis is that collection doesn’t necessitate any particular assistance [1, 23, 42] of trained medical personnel or health care professionals [2, 14]. In addition, when blood and/or urine collection [8] cannot be obtained from newborns, infants [25, 26, 38] and patients with hemorrhagic syndromes [20], supervised saliva collection is advised [1, 37]. Furthermore, patients can self-collect saliva (> 0.5 mL) without generating aerosols [22], thus greatly minimizing the risk of nosocomial virus transmission to healthcare personnel or other patients [8, 18, 25]. Saliva testing may be a useful alternative first-line screening test in several field environments, including low resource community settings [15] or in remote locations where medical facilities are lacking altogether [37]. Although largely neglected in the past [39], the diagnostic value of saliva, aided by current technological development, is expected to increase [32]. Overall, saliva could be ideal for investigating viral outbreaks [5, 13], such as COVID-19 [22, 23].

### 1.2. What can be detected in saliva?

Saliva has gradually gained wide-spread acceptance as a veritable matrix of biomarkers and other constituents ranging from organic compounds, peptides, and even microorganisms (*i*.*e*., bacteria and viruses) [1]. In addition to SARS-CoV-2 [8, 18, 35], other respiratory viruses [37], including SARS [17], dengue virus [20, 27, 40], chikungunya virus [40, 41], Epstein-Barr virus [32], HIV [6], arthropod-borne oropouche virus (OROV) [42], hepatitis A virus (HAV) [13], hepatitis C virus (HCV) [12], congenital cytomegalovirus [43], feline leukemia virus (from the domesticated cat, *felis catus*) [11], and the Zika virus [37, 44, 45], can all be detected in saliva. In fact, detection of SARS-CoV-2 viral particles in saliva up to 25 days after the onset of symptoms has been reported, supporting the potential use of saliva for monitoring viral clearance [18]. Saliva contains a variety of constituents that could also be used for the diagnosis of concurrent conditions, such as evaluation of salivary glucose and serum glucose for diabetes mellitus [46]. Other constituents have also been detected in saliva [28], such as controlled substances, sets of unique peptides [19], secretory immunoglobulin (Ig) [6, 29], proteomic biomarkers [19, 31], and breast / lung cancer-specific organic chemical signatures embedded in saliva metabolites [28].

### 1.3 Using mass spectrometry (MS) to detect viruses

Current COVID-19 tests recommended by the CDC include antigen tests and nucleic acid amplification tests (NAATs), such as PCR-based methods. However, MS-based approaches are more advantageous compared to other conventional methods. Although PCR methods have been the method of choice [47] to detect SARS-CoV-2 in the absence of symptoms due to the high degree of sensitivity,, this approach is more labor-intensive and the scope of application of the RT-qPCR assay is restricted [14]. Specifically, RT-qPCR detection of viruses is time-consuming and requires operation in a certified lab [7], trained personnel to operate the equipment, and costly kit-based reagents [5, 19, 48]. Hernandez *et al*. employed a mass spectrometric method to determine the presence of SARS-CoV-2 in human saliva [24]. MS-based methods have also been used to identify SARS-CoV-2 proteins [4] from gargle solution samples of SARS-CoV-2 patients [49]. Tomita *et al*. used capillary electrophoresis time-of-flight mass spectrometry to detect SARS-CoV-2 viral particles [30] and gel electrophoresis combined with MS was used to carry out salivary proteome separation, quantification, and identification [31]. Mass spectrometry has also been used for medical screening of cancer patients to identify cancer-specific signatures embedded in saliva metabolites [4, 30]. The traditional mass spectrometric approach is based on a conventional, non-portable, MS instrument such as MALDI-TOF that cannot directly analyze clinical samples (irrespective of sample type), as it requires sample preparation prior to analysis of viral particles in nasal or salivary secretions [3, 19]. Although, traditional MS-based methods have good sensitivity, they are relatively expensive, lab-based, and require non-portable equipment to carry out the studies [3]. In contrast, the novel MS-based SpecID methodology is a portable instrument that is capable of high-throughput, real-time, and direct analyses of a variety of sample types.

## 2. Materials and methods

### 2.1 SpecID instrumentation

SpecID is a patented, noble gas plasma spark ionization method, that can be adapted to other mass spectrometers that can be modified to sample at atmospheric pressure such as the AccuTOF (JEO USA, Inc.) or the Compact Mass Spectrometer (CMS, Advion Inc., Ithaca, NY), which was used for this project. The CMS is an atmospheric pressure ionization portable mass spectrometer, which has been modified as previously described by Alusta *et al*. [50] to perform SpecID analysis. In brief, the ionization chamber of the CMS was modified with the addition of a built-in high voltage power supply (S1A Fig) to deliver continuous sparks directly to the sample at around 2.3 keV ± keV 0.112 V via a discharge needle in an argon gas atmosphere (S1B Fig). The SpecID patented system [51–53] has been shown to ionize organic compounds, as well as microorganisms including bacteria and viruses. Sample ionization occurs when noble gas plasma sparks continuously strike analytes within a sample deposited onto depressions (*i*.*e*., indentations) of electrically grounded stainless-steel wire mesh sample holders (S2 Fig) that are electrically grounded. This type of ionization was previously described in detail in a patent [51] and four publications [50–53]. The SpecID method produces reproducible mass spectra with rich spectral information content for multiple applications including pathogens (bacteria, fungi, viruses), cream medications, solids, liquids, etc.)

### 2.2 General procedure

**Fig 1:**
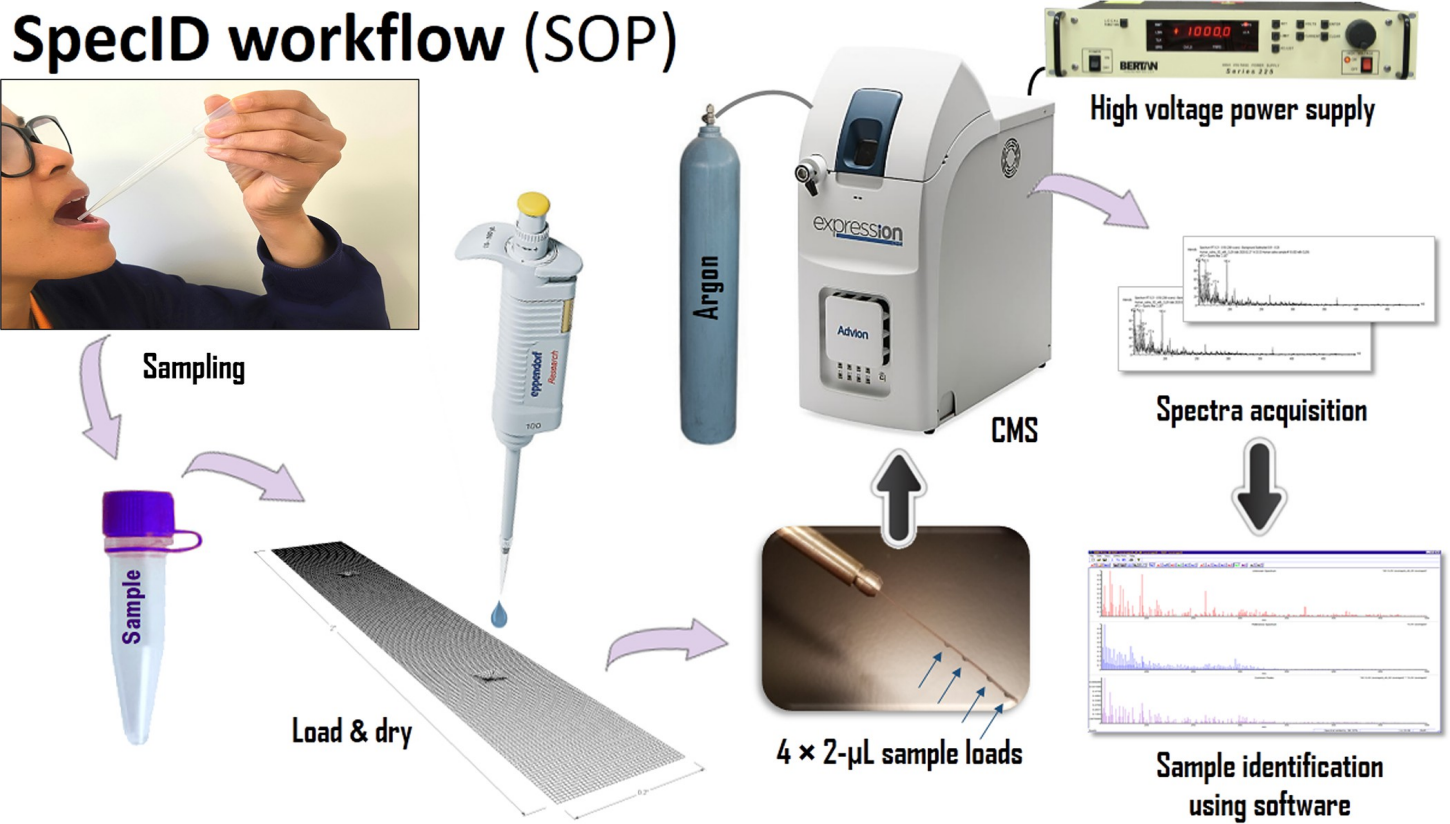
SpecID workflow (general procedure): *i*) supervised collection of saliva from a human volunteer into a 1.5 mL microcentrifuge tube, *ii*) deposition of two or more 2-μL drops of saliva on stainless-steel sample holders, *iii*) loading sample holder into the ionization chamber of the CMS, *iv*) acquisition and storage of several replicate mass spectra, *v*) extraction of replicate mass spectra from chromatogram, *vi*) analysis of presence or absence of a particular virus using spectra analysis software.

**Fig 2:**
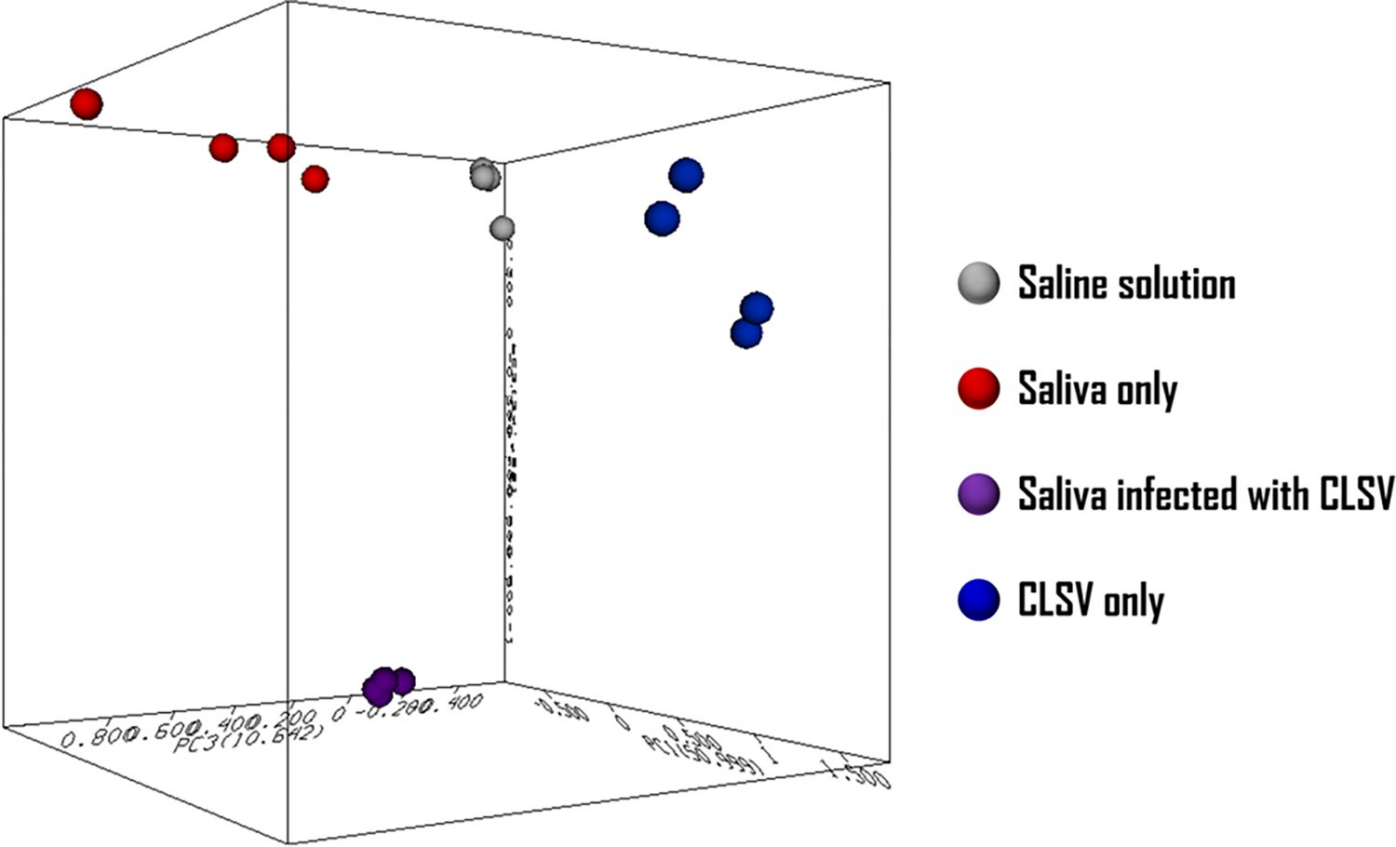
3D PCA plot: Software used: ArrayTrack^™^ (www.fda.gov/science-research/bioinformatics-tools/arraytracktm-hca-pca-standalone-package-powerful-data-exploring-tools). Notice the clusters of each replicate spectra group (saline solution, saliva only, CLSV only, and saliva treated with CLSV. This demonstrates that spectra processing with RSD can easily distinguish the samples.

The screening procedure, referred to here as the SpecID workflow for virus detection in saliva (Fig 1), consists of the following steps. Saliva was collected in 1.5 mL microcentrifuge tubes (on average 0.4 to 0.5 mL), and vigorously vortexed for 10–15 s to ensure homogeneity. As many as four 2-μL replicate samples of saliva were transferred by pipette to a sample holder. Sample holders (S2 Fig) were inserted into the ionization chamber of the CMS using a probe (S1A Fig) where they were spark-ionized, as described above. The duration of the spark ionization and detection process was approx. 8–10 s per sample. The mass spectra (ranging from 151 to 500 amu) were collected and stored using the CMS software supplied by the manufacturer. The replicate mass spectra of each sample on the sample holder were acquired and used by post spectral processing software to improve the signal and reduce the noise in the scans. Replicate mass spectra were later extracted from the respective total ion chromatogram (TIC) using the CMS data processing software. Spectra were processed, as described below, and each sample’s unique viral spectral silhouette was analyzed using 3D principal component analysis (PCA) plots (Fig 2).

### 2.3 Virus and saliva samples

Human saliva was obtained from consenting volunteers from August 1, 2021, to October 31, 2021 (FDA IRB approved project). The volunteer saliva was spiked with cucumber leaf spot virus (CLSV, S3A Fig, provided by Michael Sherman, University of Texas Medical Branch at Galveston, TX) at a concentration of approximately 10^6^ viral particles / mL of saliva, which corresponds to the approximate viral load of SARS-CoV-2 in a COVID-19 patient [54]. The canine coronavirus (CoV) suspension was obtained from our co-author Marli Azevedo (NCTR Microbiology Division) and stored in HEPES (1.5 mM Na_2_HPO_4_·2H_2_O aqueous solution) buffer. Similarly, human coronavirus (HCoV) OC43 (S3B Fig) viral suspension, HCoV NL63 viral suspension, and bovine coronavirus (BCoV) were obtained from Dr. Azevedo. In addition, Dr. Azevedo also provided us with heat inactivated SARS-CoV-2 viral strains used in this project, which were obtained from *bei RESOURCES* (www.beiresources.org).

### 2.4 Resolution and Limit of Detection (LOD)

**Fig 3:**
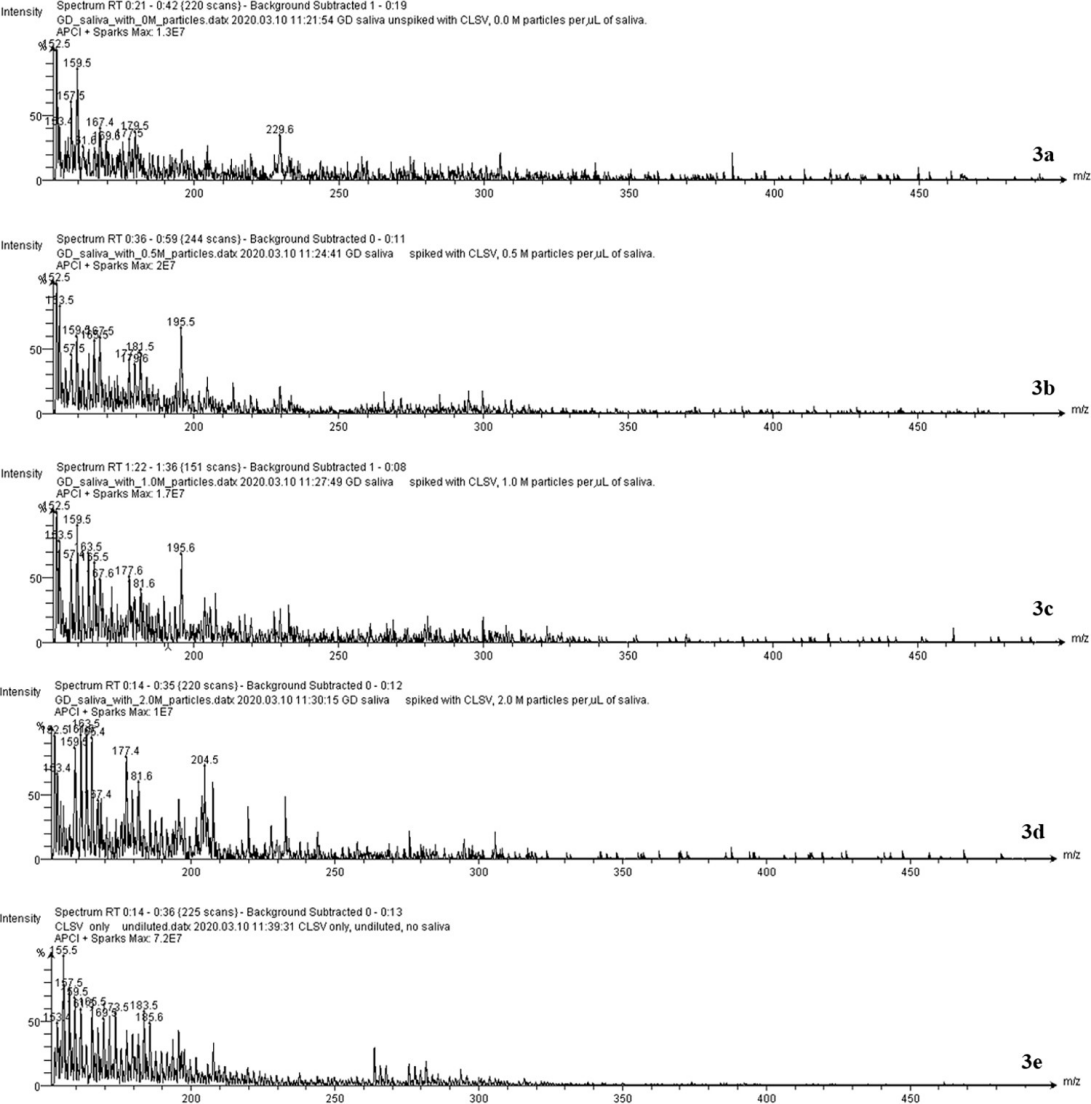
Spectra comparison of human saliva spiked with CLSV at various concentrations. From top to bottom: saliva only (a), 0.5 M viral particles per mL of saliva (b), 1.0 M viral particles per mL of saliva (c), 2.0 M viral particles per mL of saliva (d), and finally CLSV only (e).

**Fig 4:**
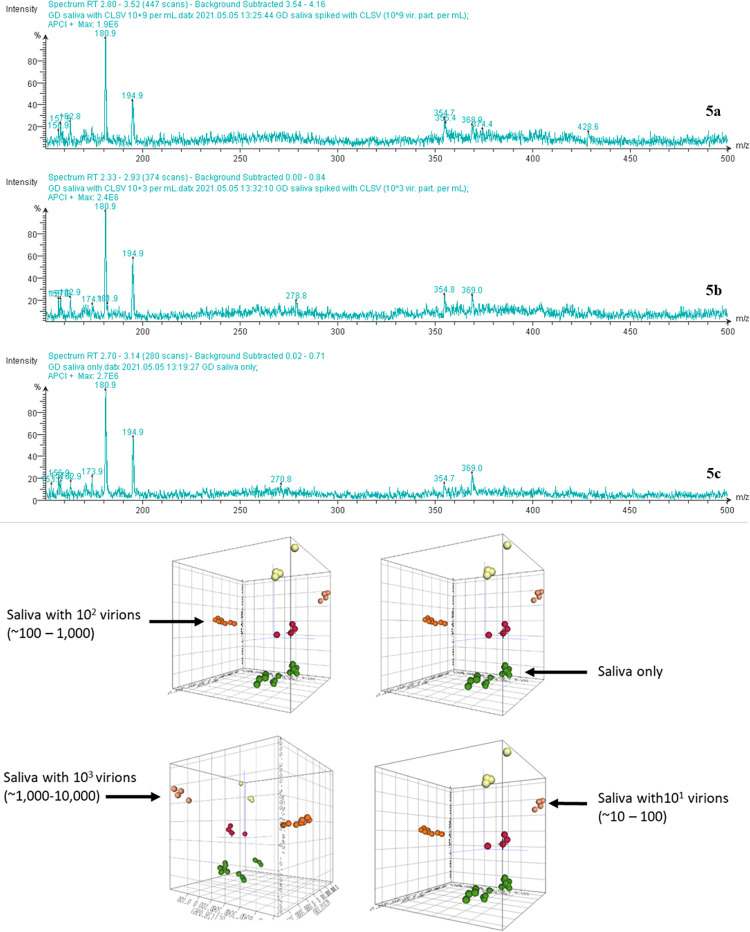
Determination of limit of detection (LOD): From top to bottom: human saliva spiked with 10^9^ virions / mL (a), human saliva spiked with 10^3^ virions / mL (b), human saliva only (c), 3D PCA plot showing distinct clusters of human saliva spiked with viral loads of 0, 10^1^, 10^2^, and 10^3^ virions (d). Notice the clear-cut cluster separations in (d).

To determine if viruses can be detected at low levels in human saliva, replicate spectra of 1) CLSV only, 2) saliva only, and 3) CLSV-spiked human saliva were acquired on a SpecID platform. To determine the lower limit of detection, mass spectra of human saliva were spiked with CLSV at various concentrations (Fig 3A–3E) of serial dilutions, ranging from 0 to 2.0 × 10^6^ viral particles/mL, and spectra were acquired using the SpecID platform and processed, as described above. The LOD was determined to be <10^3^ virions / mL (Fig 4A–4D). Processed spectra were plotted as respective spheres using a 3D PCA plot (Fig 2). Clusters of spheres within each group were evident in the PCA plot, demonstrating that replicate spectra (acquired from the same sample) obtained from CLSV-spiked human saliva (Fig 3D and 3E) were identified and distinguished from the replicate samples (Fig 3A) obtained from human saliva of a healthy individual.

### 2.5. Spectra processing

**Fig 5:**
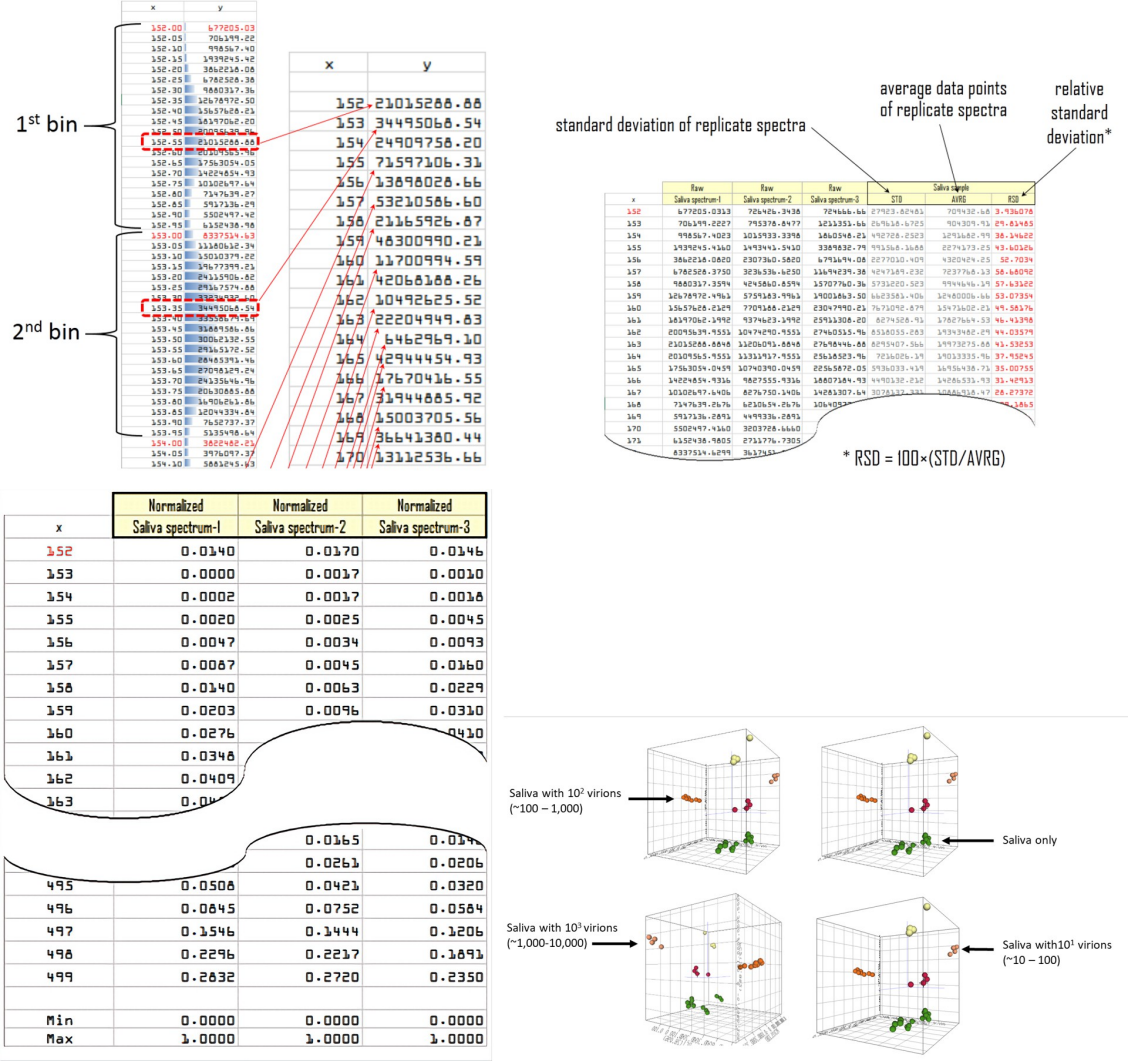
a: Spectral binning: Illustration of binning mass spectra stored as Cartesian coordinates arranged in a spreadsheet. Individual spectra data points are binned at a resolution of 1 amu. Only the maximum value (red dotted line) found within each bin is adopted for each bin (red arrows). Analysis of binned spectra is much quicker due to the file size, when compared to raw data. b: Noise reduction: Division by Relative Standard Deviation (RSD) to reduce background noise. Each spectral data point is divided by the relative standard deviation spectrum (calculated from the respective sample replicates). c: Spectral normalization: To facilitate spectral analysis, the amplitudes of each spectrum are shifted between 0.000 and 1.000, as calculated by their minima and maxima (see bottom of table shown in c).

Extracted raw spectra data files are fairly large, requiring extensive storage disc space (ca. 2 MB ea.) on electronic media. For this reason, the raw spectra files were binned to 1 amu (see Fig 5A for details) to reduce their file size, such that 1 bin encompassed spectral features within each amu. Acquired mass spectra contained a random nominal amount of background noise, which is a natural occurrence during spectral acquisition of biological samples or organic compounds due to electronic and/or mechanical noise. This noise can negatively impact spectral reproducibility for data analysis, so processing to reduce or eliminate the noise is necessary. An RSD noise reduction algorithm was developed to reduce background noise, wherein binned spectra were divided by their respective relative standard deviation (*i*.*e*., RSD, see Fig 5B for details), which was calculated from a set of 4–6 spectral replicates for each sample on a peak-by-peak basis. Spectra were then normalized between 0 and 1 (Fig 5C) to facilitate the use of spectra for data analysis and spectra comparison.

## 3. Results

### 3.1. Classifying coronaviruses

**Fig 6:**
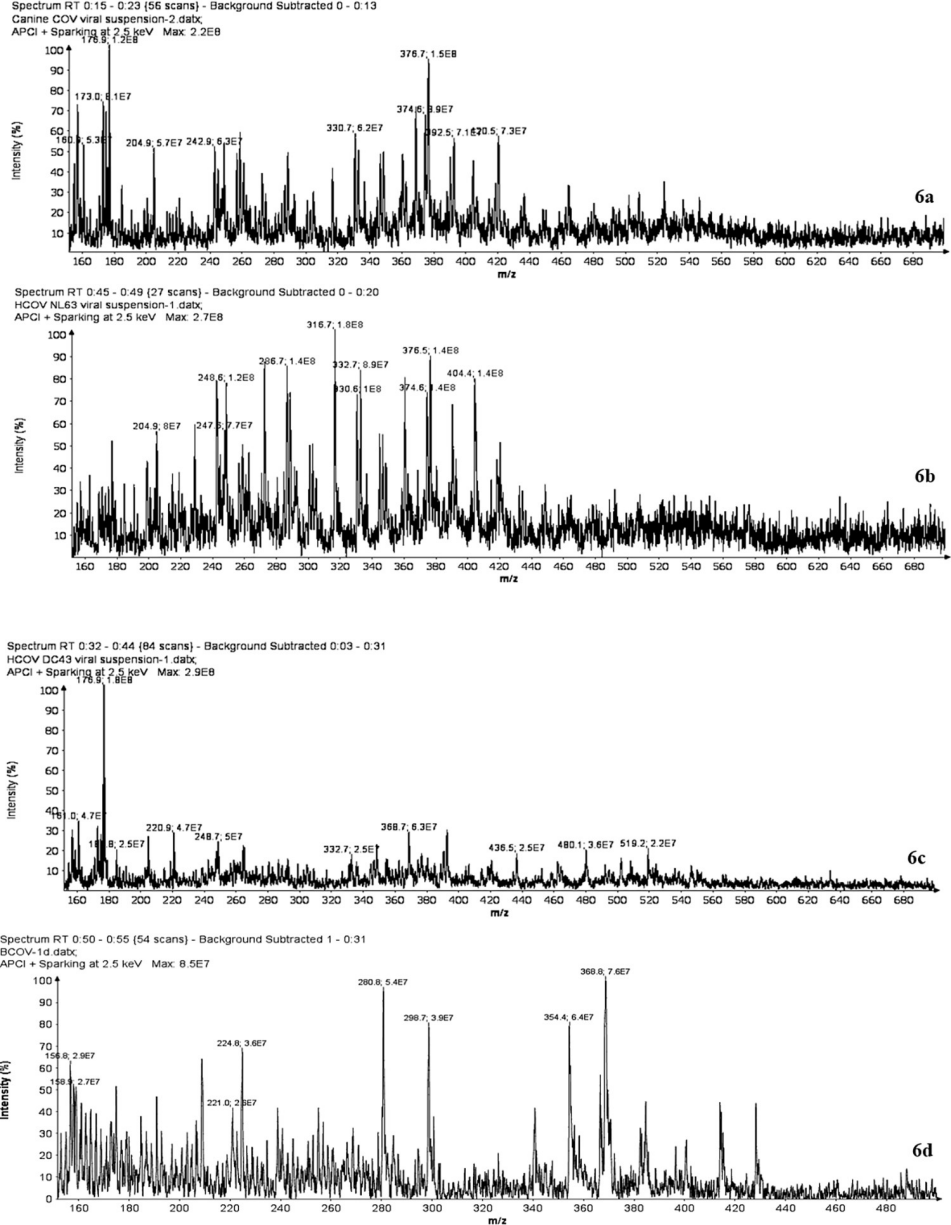
Comparison of mass spectra acquired of canine CoV (a), HCoV NL63 (b), HCoV OC43 (c), and bovine coronavirus Mebus (d). Distinctive spectral features can be easily identified by means of visual inspection.

In addition to determining the LOD for the SpecID platform, it was also important to determine the platform’s ability to distinguish genetically homologous viruses. To this end, SpecID mass spectra were acquired for four coronaviruses, specifically 1) a canine CoV viral suspension in HEPES (1.5 mM Na_2_HPO_4_ · 2H_2_O aqueous solution) buffer, 2) a HCoV OC43 (S3C Fig) viral suspension in buffer, 3) a HCoV NL63 viral suspension in buffer, and 4) bovine coronavirus Mebus (BCoV Mebus) in HEPES buffer (S3B Fig). HCoV OC43 and BCoV Mebus were chosen for analysis because they have >95% genetic homology. By looking at a side-by-side visual comparison of the mass spectra acquired from canine CoV (Fig 6A), HCoV NL63 (Fig 6B), HCoV OC43 (Fig 6C) and bovine coronavirus Mebus (Fig 6D) unique reproducible spectral features could be observed for each virus. When analyzed using the PCA software, the viruses were separated into distinctive clusters of replicates. Importantly, this demonstrated that SpecID was capable of distinguishing various coronaviruses, even ones with close genetic homology.

### 3.2. Distinguishing SARS-CoV-2 variants

The ability of the SpecID platform to differentiate SARS-CoV-2 variants was also investigated. Human saliva was spiked with heat-inactivated delta variant (NR-56128, SARS-CoV-2 isolate hCoV-19/USA/MD-HP05285/2021, lineage B.1.617.2) or omicron variant (NR-56495, SARS-CoV-2 isolate hCoV-19/USA/GA-EHC-2811C/2021, lineage B.1.1.529) and SpecID mass spectra were obtained.

**Fig 7:**
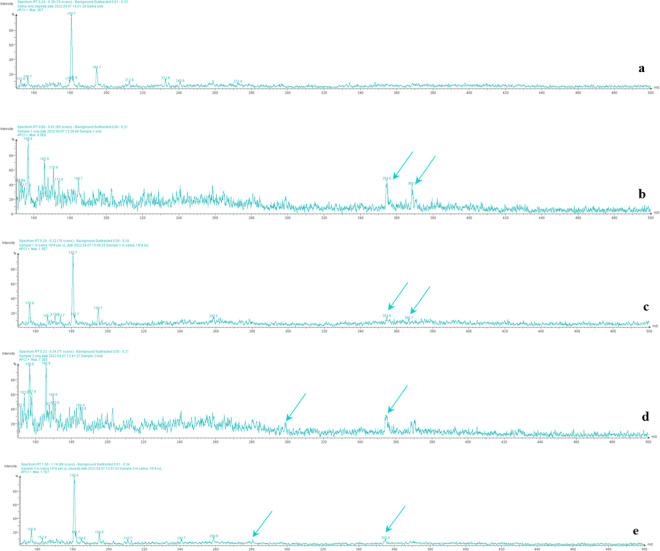
Spectra of human saliva spiked with the SARS-CoV-2 variants delta and omicron. From top to bottom: human saliva only (a); NR-56128, heat-inactivated SARS-CoV-2, hCoV-19, B.1.617.2, delta variant, 10^4^ virons suspended in 1 mL of PBS (b); NR-56128, heat-inactivated SARS-CoV-2, hCoV-19, B.1.617.2, delta variant, 10^4^ viral particles suspended in 1 mL of human saliva (c); NR-56495, heat-inactivated SARS-CoV-2, hCoV-19, B.1.1.529, omicron variant, 10^4^ viral particles suspended in 1 mL of PBS (d); NR-56495, heat-inactivated SARS-CoV-2, hCoV-19, B.1.1.529, omicron variant, 10^4^ viral particles suspended in 1 mL of human saliva (e). The arrows point to peaks of metabolites that aid visual confirmation.

**Fig 8:**
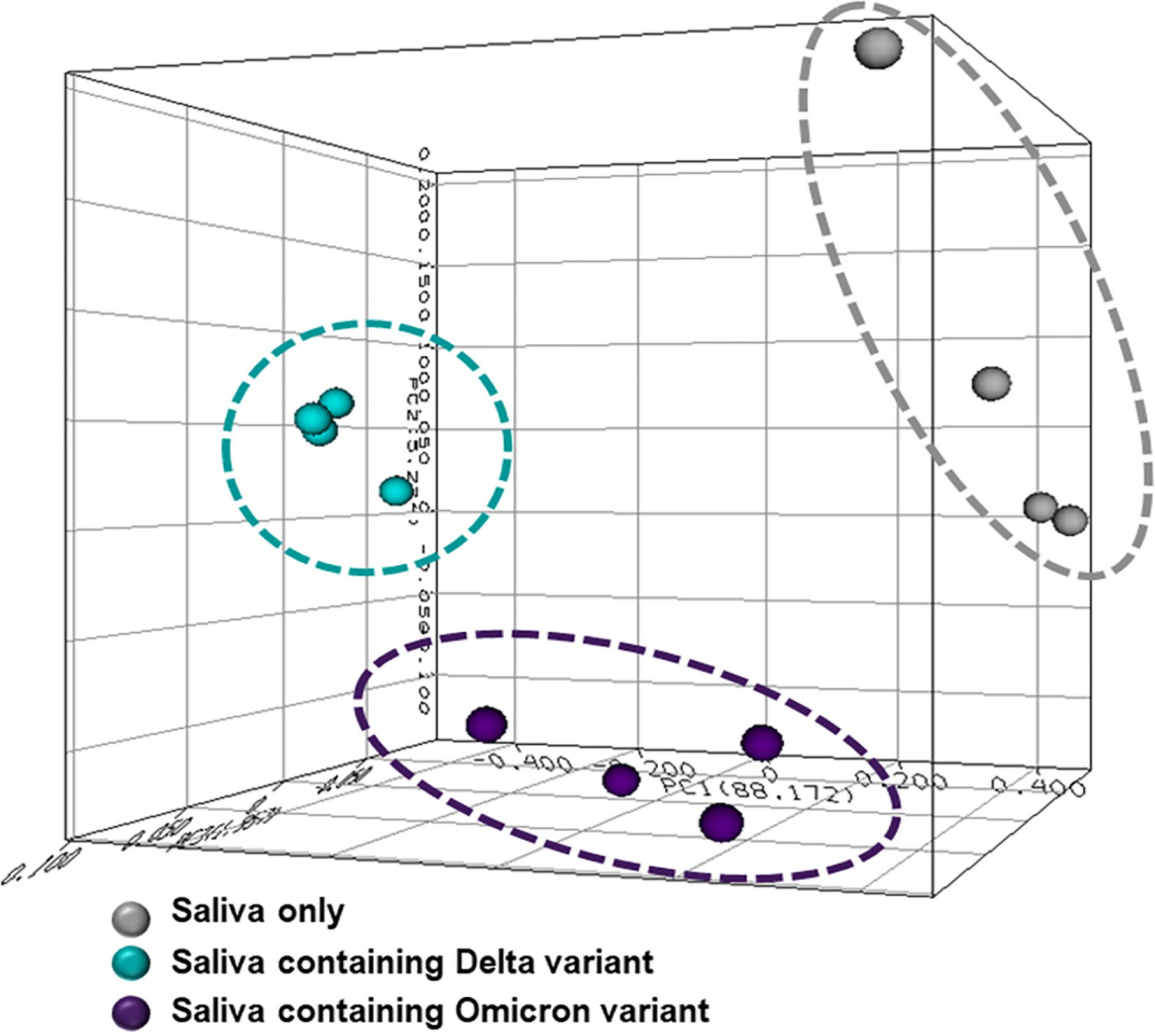
3D PCA of spectra of human saliva only, and spectra of saliva spiked with 500 virions of delta and omicron variants. Notice the spatial clustering of each group, demonstrating that spectral distinction of these variants can be achieved with SpecID at this low level of virus in saliva.

Mass spectra peaks associated with delta (Fig 7B) and omicron (Fig 7D) viral stock solutions were also identified in human saliva spiked separately with delta (Fig 7C) and omicron SARS-CoV-2 variants (Fig 7E), which are distinguishable from mass spectra acquired from samples of human saliva only (Fig 7A). Reproducible prominent peaks of the spark ionization break down products (arrows) were identified for both variants. Furthermore, 3D PCA of spectra acquired from human saliva, compared to the processed mass spectra acquired of human saliva spiked with the SARS-CoV-2 variants indicates spatial clustering for the two variants, delta and omicron (Fig 8). The spatial clustering of each group of replicate spectra in the plot illustrates this point. This was possible using the RSD noise reduction algorithm developed ‘in-house’ by NCTR.

## 4. Discussion

**Fig 9:**
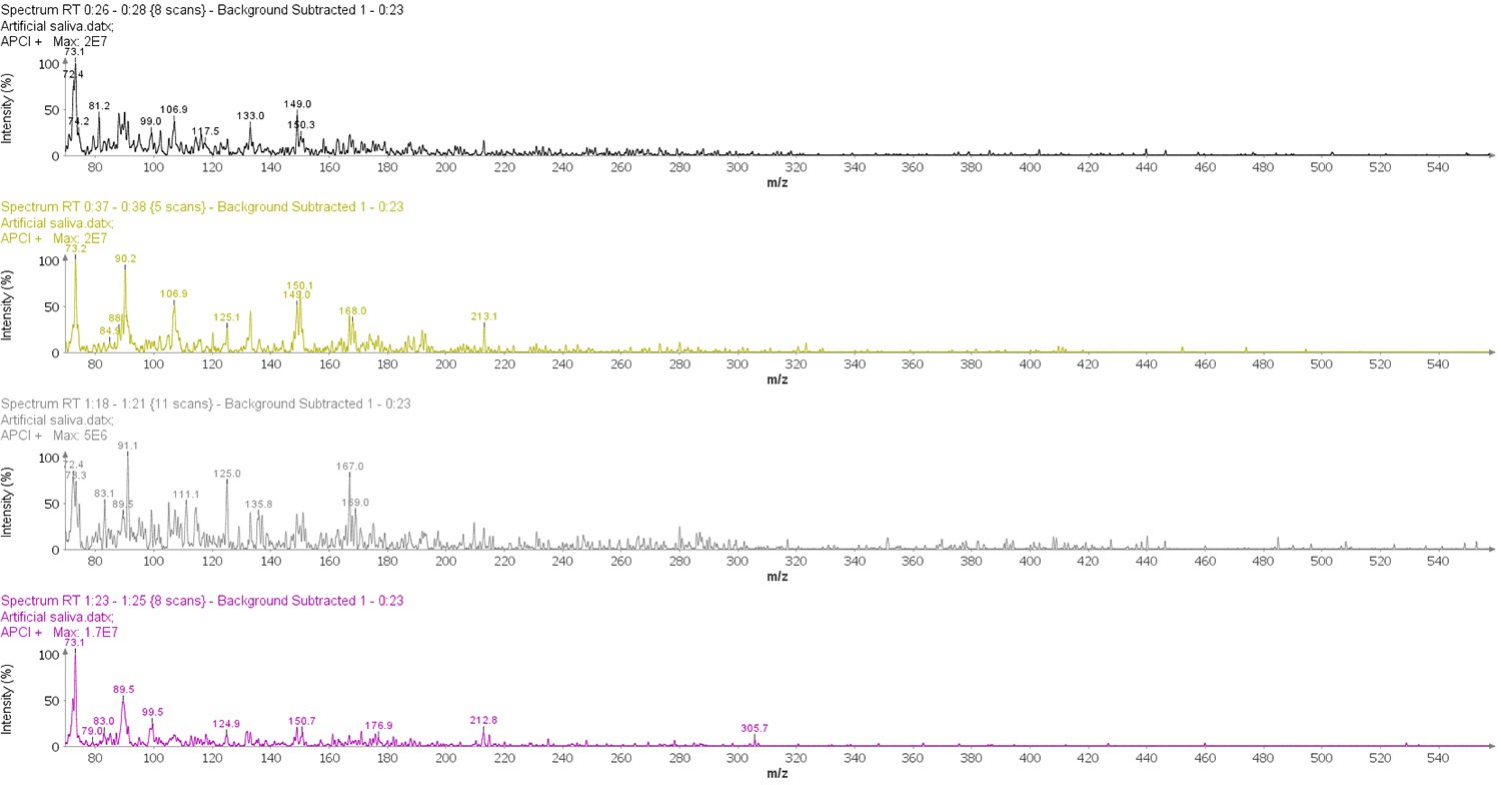
Spectra of artificial saliva demonstrating that it could not be used for analysis due to lack of reproducibility from on sample to the next.

The SpecID technology was discovered while working with bacteria and has since been demonstrated for multiple applications [50, 52] and for multiple sample types. Furthermore, saliva is an easily collected specimen and a good source for biological indicators, ranging from alterations in nucleic acids, proteins, and microflora biochemicals [55], which could convey good potential, compared to other biological fluids, for use in the detection of respiratory viruses using SpecID. Thus, SpecID was investigated for the potential to rapidly detect viruses and strain variants, which could be useful in monitoring viral outbreaks, such as the COVID-19 pandemic. Since Covid-19 is a respiratory virus, and saliva is a route of transmission, saliva was a logical matrix for analysis using SpecID. The initial SpecID work using CLSV virus in saliva, as a surrogate for Covid, showed that: 1) saliva spectra are reproducible, 2) saliva containing virus could easily be distinguished from saliva only samples, and 3) viral load in the saliva can easily be detected. Limit of detection experiments showed that the SpecID sensitivity for detection of viruses in saliva was similar to PCR, but that the time for data acquisition was less than 1 minutes, including loading of the sample into the instrument, as compared to ≥ 2 hours for RT-PCR. Once an automated viral database is created, the database will be able to provide information on viruses or variants in the database. In the case of a new variant, the spectrum will be unique and identified as such by the database. The sample will be kept for genetic analysis and once genetic analysis confirms the new variant, the label can be placed on all the spectra collected with that unique signature. However, even though initially there is no label for a new variant, the SpecID unique signature for the variant will still identify the infected individuals while the genetic assay is being performed. Artificial saliva (Pickering Laboratories, Mountain View, CA) was initially tested as a preliminary surrogate to human saliva. It is a mixture of chemicals that approximate the viscosity and some of the physical characteristics of saliva. It was tested for use with SpecID, and the replicate spectral patterns were not reproducible and reliable compared to human donor saliva (Fig 9). For this reason, artificial saliva was not used. The saliva used in this study was collected from consenting healthy human volunteers under oversight and approval of the FDA IRB, and used to prepare the various spiked viral suspensions that were analyzed using the SpecID. Volunteers who agreed to participate in our study, provided saliva in 1.5 mL microcentrifuge tubes at least 60 min after the most recently ingested meal, in some instances, after teeth brushing. Volunteers were informed that consumed products can appear in saliva, and this includes recent consumption of food, soft drinks, alcoholic beverages, smoking, vaping, smokeless tobacco products, chewing bubble gum, and the presence of controlled substances (Schedules I-V) in trace amounts. Given the sensitivity of the SpecID technology, the presence of adulterants or some health conditions that affect saliva composition may also be detected in saliva spectra. Examples of health conditions that may be detectable via SpecID could include dehydration, reflux disease, poor hygiene, and other medical conditions such as decaying teeth, tonsillitis, bronchitis, benign lymphoepithelial lesions [56], and diabetes. Thus, establishing archived SpecID spectra of food, drugs, other chemicals, disease states, and other adulterants in a database could be useful as historical controls of virus free saliva by enabling background subtraction and facilitating identification of viruses in a variety of background conditions. Identifying a specific virus in saliva during high-throughput screening of the general population can be challenging, as people will most likely exhibit some variability in their saliva, however, the SpecID system was sufficiently sensitive that it was able to deconvolute the background noise from different saliva donors and accurately identify viral particles at low concentrations. This suggests that identification by comparison to catalogued viral spectral fingerprints could be possible in saliva with food ingested or other consumed substances and medical conditions. A software program was developed to extract background spectra from the total ion chromatogram, then process the spectra for noise reduction and comparison. This approach was used to successfully identify viruses and distinguish viral strains in human saliva.

## 5. Conclusions

Compared to other body fluids (*e*.*g*., blood), collection and testing of saliva is painless and enables rapid screening of infected samples. Compared to sampling of other body fluids such as blood and urine, the SpecID workflow for saliva is easy to perform. There is no sample preparation and saliva samples can be easily collected and introduced directly into the mass spectrometer’s ionization chamber using a sample holder. Replicate spectra are acquired in less than a minute and with the development of an automated database, the spectra could be quickly extracted, RSD-processed, and analyzed, which could be obtainable in seconds with the addition of software automation. Virus identification, even at the variant-level, could be accomplished by interrogating a library or database of spectral fingerprints using automated software. In this study, cucumber leaf spot virus (CLSV) was detected and identified in spiked human in saliva vs. saliva only, with a LOD of 10^3^ virions / mL; various coronaviruses were distinguished in spiked human saliva vs. saliva-only control, and the SARS-CoV-2 variants (delta and omicron) were distinguished in human saliva samples. The SpecID RSD processing and analysis software was necessary to produce the results, which could be automated, as mentioned above. The use of the SpecID platform as discussed in this manuscript consists of 1) the instrument modifications that produces argon plasma spark ionization, and 2) the replicate-based RSD processing software. This system could be extended to the rapid and reliable detection and identification of other clinically significant viruses such as Zika, dengue, influenza, hepatitis B and C [10, 56], human immunodeficiency virus (HIV), and many others including newly emerging viruses. For this reason, the SpecID platform could be extremely useful in clinical settings and for national biodefense purposes. In addition to clinical testing of saliva, the platform could be expanded for testing with other body fluids and other matrices (e.g. air, water, surface wipes) relevant for environmental testing for viruses in public places such as airports, subway train stations, product testing labs in regulatory environments, quality control departments in industrial production facilities, and even possibly more sites. The portability of the SpecID system enables sample analysis in the field ‘as is’ (no sample prep), without the need for reagents (a kit). Examples of other assays performed so far include 1) medications—solids, gels, creams, liquids, 2) supplements, 3) food–solid, liquid, fat, emulsion, etc., 4) biofluids and solids, and many other matrices, and 5) bacteria (strain level). The SpecID platform has many other potential uses, and our plan is to highlight those applications which will be highlighted in future publications.

## Supporting information

S1 Fig a) The SpecID modified ionization chamber of the Advion CMS mass spectrometer. The sample holder (dark gray rod and yellow aggregate) is depicted being inserted in the chamber. All CAD drawings reproduced in this study were made by the authors using SketchUp vers. 14. b) Cut-through view of the modified ionization chamber of the CMS. The tip of the discharge needle (D) is located right above the ion inlet orifice (I)–top of the cone.
(ZIP)

S2 Fig Low-magnification SEM micrograph of the stainless-steel wire mesh “sample holder”. The up-side-down indentation which can hold up to 5 μL of saliva ready to be ionized.
(TIF)

S3 Fig a) TEM micrograph (magnification: 40,000 ×) of cucumber leaf spot virus (CLSV) virions. The average diameter of a CLSV virion is ~30 nm. b) TEM micrograph (magnification: 25,000 ×) of bovine coronavirus (BCOV). The average diameter of BCOV virus is ~90 nm. c) TEM micrograph (magnification: 25,000 ×) of HCOV OC43 virions. The average diameter of a HCOV OC43 virus is ~95 nm.
(ZIP)

S1 Data Analysis COVID Variants 500 virions per mL v1.0. (TXT)

S2 Data Canine HCOV vs buffer time analysis. (TXT)
