# Supplementary figures and images for: Direct detection and identification of viruses in saliva using a SpecID ionization modified mass spectrometer
Source: PLoS One. 2025 Feb 7;20(2):e0316368. doi: 10.1371/journal.pone.0316368 (PMC11805448; doi:10.1371/journal.pone.0316368)

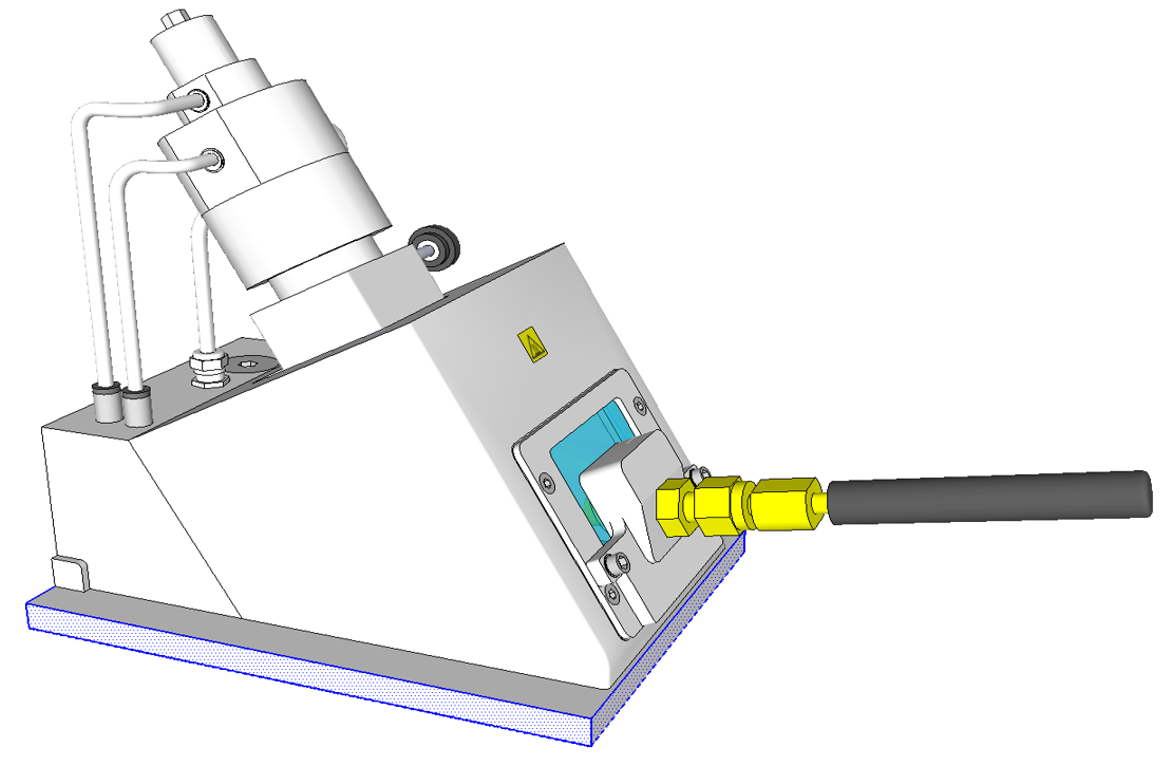

Supplement: S1 Fig — a) The SpecID modified ionization chamber of the Advion CMS mass spectrometer. The sample holder (dark gray rod and yellow aggregate) is depicted being inserted in the chamber. All CAD drawings reproduced in this study were made by the authors using SketchUp vers. 14. b) Cut-through view of the modified ionization chamber of the CMS. The tip of the discharge needle (D) is located right above the ion inlet orifice (I)–top of the cone. (ZIP) [file pone.0316368.s001.zip › S1a_Fig.tif]

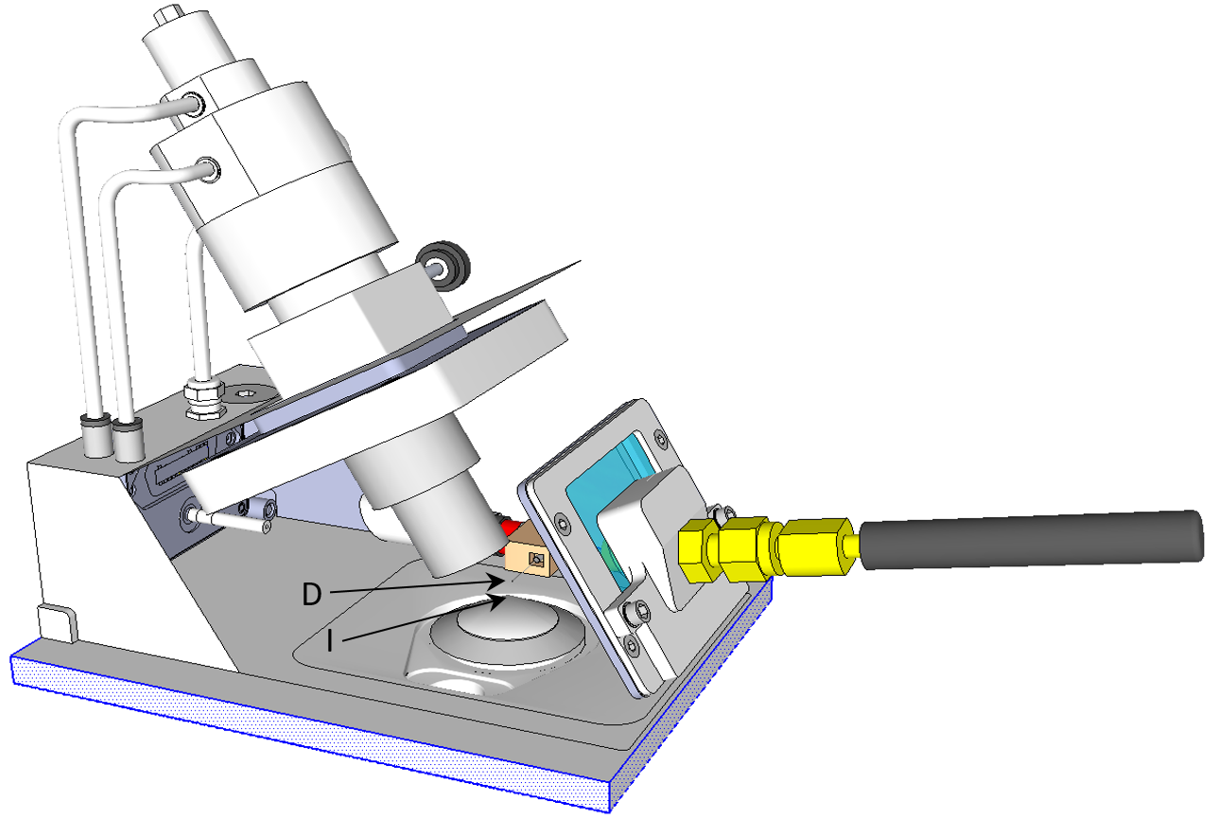

Supplement: S1 Fig — a) The SpecID modified ionization chamber of the Advion CMS mass spectrometer. The sample holder (dark gray rod and yellow aggregate) is depicted being inserted in the chamber. All CAD drawings reproduced in this study were made by the authors using SketchUp vers. 14. b) Cut-through view of the modified ionization chamber of the CMS. The tip of the discharge needle (D) is located right above the ion inlet orifice (I)–top of the cone. (ZIP) [file pone.0316368.s001.zip › S1b_Fig.tif]

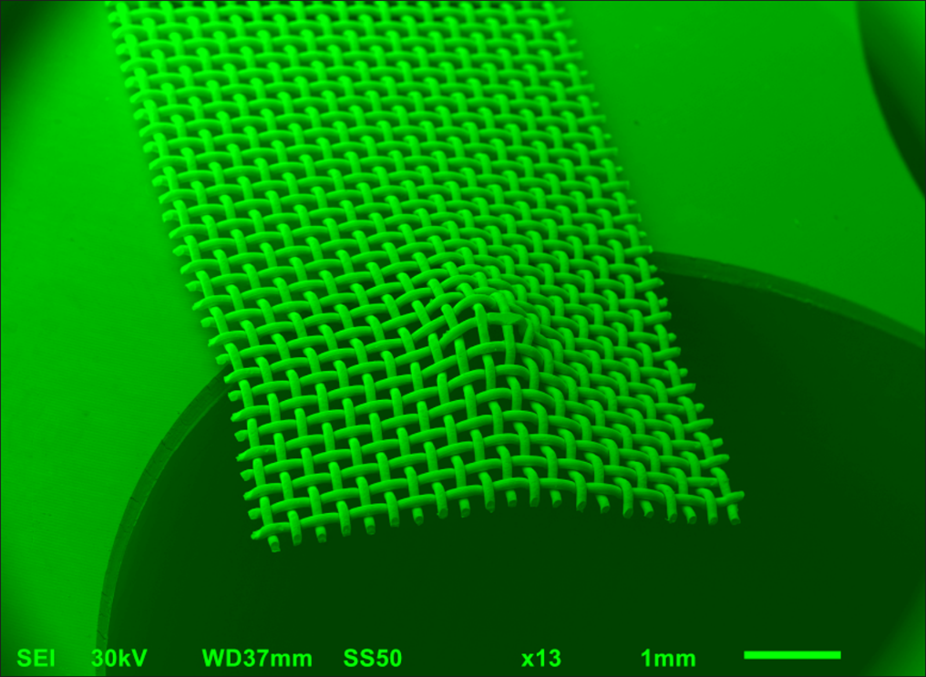

Supplement: S2 Fig — The up-side-down indentation which can hold up to 5 μL of saliva ready to be ionized. (TIF) [file pone.0316368.s002.tif]

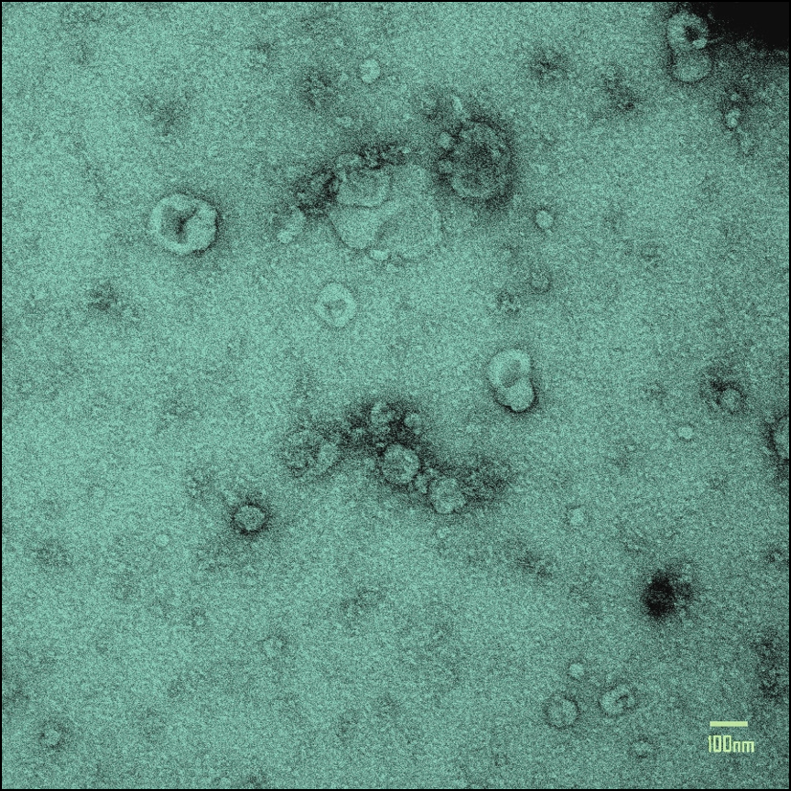

Supplement: S3 Fig — a) TEM micrograph (magnification: 40,000 ×) of cucumber leaf spot virus (CLSV) virions. The average diameter of a CLSV virion is ~30 nm. b) TEM micrograph (magnification: 25,000 ×) of bovine coronavirus (BCOV). The average diameter of BCOV virus is ~90 nm. c) TEM micrograph (magnification: 25,000 ×) of HCOV OC43 virions. The average diameter of a HCOV OC43 virus is ~95 nm. (ZIP) [file pone.0316368.s003.zip › S3b_Fig.tif]

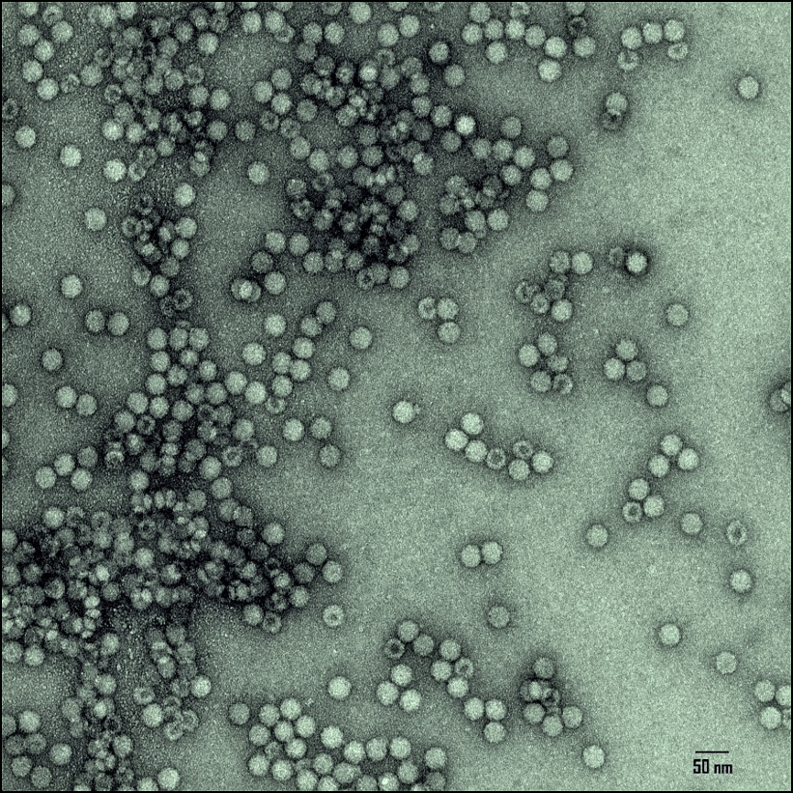

Supplement: S3 Fig — a) TEM micrograph (magnification: 40,000 ×) of cucumber leaf spot virus (CLSV) virions. The average diameter of a CLSV virion is ~30 nm. b) TEM micrograph (magnification: 25,000 ×) of bovine coronavirus (BCOV). The average diameter of BCOV virus is ~90 nm. c) TEM micrograph (magnification: 25,000 ×) of HCOV OC43 virions. The average diameter of a HCOV OC43 virus is ~95 nm. (ZIP) [file pone.0316368.s003.zip › S3a_Fig.tif]

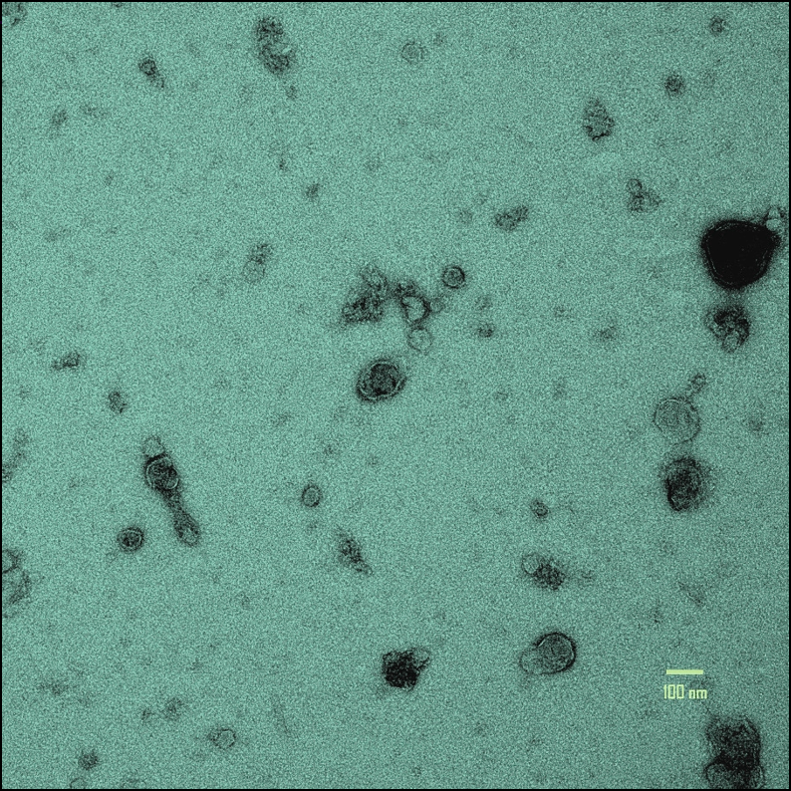

Supplement: S3 Fig — a) TEM micrograph (magnification: 40,000 ×) of cucumber leaf spot virus (CLSV) virions. The average diameter of a CLSV virion is ~30 nm. b) TEM micrograph (magnification: 25,000 ×) of bovine coronavirus (BCOV). The average diameter of BCOV virus is ~90 nm. c) TEM micrograph (magnification: 25,000 ×) of HCOV OC43 virions. The average diameter of a HCOV OC43 virus is ~95 nm. (ZIP) [file pone.0316368.s003.zip › S3c_Fig.tif]
